# Morphological and molecular identification of two new wood-inhabiting species (*Russulales*, *Basidiomycota*) from China

**DOI:** 10.3897/mycokeys.131.192179

**Published:** 2026-04-27

**Authors:** Changlin Zhao, Qi Li, Xiangfu Liu

**Affiliations:** 1 Modern Industry School of Edible-fungi, Southwest Forestry University, Kunming 650224, China Department Microbial Drugs (MWIS), Helmholtz Centre for Infection Research Braunschweig Germany; 2 Department Microbial Drugs (MWIS), Helmholtz Centre for Infection Research, Inhoffenstraße 7, 38124 Braunschweig, Germany College of Forestry, Southwest Forestry University Kunming China; 3 College of Forestry, Southwest Forestry University, Kunming 650224, China Modern Industry School of Edible-fungi, Southwest Forestry University Kunming China; 4 College of Biological Science and Food Engineering, Southwest Forestry University, Kunming 650224, China College of Biological Science and Food Engineering, Southwest Forestry University Kunming China

**Keywords:** Fungal diversity, molecular systematics, new taxa, taxonomy, Yunnan Province

## Abstract

Wood-inhabiting fungi represent a highly diverse group of microorganisms that play essential roles in forest ecosystems. However, the species diversity of wood-inhabiting fungal taxa in the order *Russulales* has not been systematically documented in the high-altitude forests of Yunnan Province, China. In the present study, the detailed morphological characteristics and combined multi-locus phylogeny of the internal transcribed spacers (ITS) and the large subunit (nrLSU) of the nuclear ribosomal DNA (rDNA) revealed two new species in *Russulales*, viz., *Gloeopeniophorella luteola* and *Scytinostroma tenuissimum*. *Gloeopeniophorella luteola* is characterized by its membranaceous, thin basidiomata with a smooth hymenial surface, a monomitic hyphal system with simple septa, generative hyphae, and gloeocystidia, and thick-walled basidiospores measuring 4–4.9 × 3.8–4.4 µm. *Scytinostroma tenuissimum* is characterized by its soft membranaceous basidiomata with a cream to pale pink hymenial surface, a dimitic hyphal system with clamped generative hyphae, and ellipsoid basidiospores measuring 10.1–13.2 × 7.1–9.3 µm. Phylogenetic analysis based on the concatenated ITS + nLSU dataset demonstrated that the two new species grouped within the order *Russulales*, in which *G. luteola* grouped into the family *Russulaceae*, while *S. tenuissimum* grouped into the family *Peniophoraceae*, and *G. luteola* was sister to *G. bambusicola*, and *S. tenuissimum* grouped together with *S. acystidiatum* and *S. bambusinum*. Comprehensive descriptions, micrographs, and phylogenetic analyses of the two new species are provided.

## Introduction

Fungi are eukaryotic microorganisms that play key ecological roles as decomposers, mutualists, or pathogens (Hibbett et al. 2025). The classification of the fungal kingdom has been continuously updated, with frequent inclusion of data from DNA sequences and both fresh material and cultures. Wood-inhabiting fungi are considered “key players” in wood decomposition because of their ability to produce lignocellulosic enzymes that break down woody lignin, cellulose, and hemicellulose (Tedersoo et al. 2014; Case et al. 2025).

The order *Russulales* contains not only mushroom taxa but also wood-inhabiting fungi, e.g., *Asterostroma* Massee, *Baltazaria* Leal-Dutra, Dentinger & G.W. Griff., *Gloeopeniophorella* Rick, *Hericium* Pers., *Heterobasidion* Bref., *Peniophora* Cooke, *Scytinostroma* Donk, and *Vararia* P. Karst. (Miller et al. 2006; Deng et al. 2024). Over the past decades, many new taxa of *Russulales* have been documented using integrative approaches combining morphology and DNA-based phylogenetics (Wu et al. 2020; Zou et al. 2022; Deng et al. 2024; Dong et al. 2025). Resupinate basidiomata play a key role in several families of this order, such as *Echinodontiaceae* Donk, *Peniophoraceae* Lotsy, *Russulaceae* Lotsy, and *Stereaceae* Pilát (Wu et al. 2020; He et al. 2024; Wang et al. 2025a, b).

The genus *Gloeopeniophorella* was proposed by Rick (1934), with *G. rubroflava* Rick as the type species. This genus is characterized by resupinate basidiomata, a smooth to tuberculate hymenophore surface, a monomitic hyphal system with simple-septate generative hyphae, and ellipsoid basidiospores (Bernicchia and Gorjón 2010). Phylogenetic relationships of russuloid basidiomycetes, with emphasis on aphyllophoralean taxa, showed that *Gloeopeniophorella convolvens* (P. Karst.) Boidin, Lanq. & Gilles and *G. laxa* (Sheng H. Wu) Boidin, Lanq. & Gilles closely grouped and then clustered in *Russulales* with a smooth hymenophore (Larsson and Larsson 2003). Re-thinking the classification of corticioid fungi revealed that two genera, *Gloeopeniophorella* and *Lactarius*, grouped together and then nested within the family *Russulaceae* (Larsson 2007). The phylogeny of *Gloeopeniophorella* based on combined ITS + nLSU sequence data revealed that this genus was nested in *Russulaceae*, in which three species were grouped into this genus (Yang et al. 2025).

The genus *Scytinostroma* Donk was established by Donk (1956) and typified by *S. portentosum* (Berk. & M.A. Curtis) Donk, which has resupinate, coriaceous basidiomata; a smooth to tuberculate hymenophore; a dimitic hyphal system with simple septa or clamps on generative hyphae; skeletal hyphae densely branched and sometimes forming dendrohyphae or dichohyphae, strongly dextrinoid and cyanophilous; the presence of cystidia; basidia tubular to uniform and subglobose to ellipsoid, smooth, thin-walled, and variably amyloid basidiospores; and causes white rot (Donk 1956; Bernicchia and Gorjón 2010; Tabish and Daniel 2021). Currently, 63 specific and infraspecific names are recorded in Index Fungorum and MycoBank (accessed 18 March 2026), of which 44 are accepted worldwide (Donk 1956; Bernicchia and Gorjón 2010; Dong et al. 2024). Phylogenetic research on *Scytinostroma* revealed that the taxa *S. galactinum* (Fr.) Donk, *S. jacksonii* Boidin, *S. ochroleucum* Donk, *S. odoratum* (Fr.) Donk, and *S. portentosum* (type species) were clustered in five different, distantly separated lineages in *Peniophoraceae* (Larsson and Larsson 2003; Miller et al. 2006). Leal-Dutra et al. (2018) conducted phylogenetic research to reveal the phylogenetic relationships among *Scytinostroma* taxa and proposed a new genus, *Baltazaria* Leal-Dutra, Dentinger & G.W. Griff., to accommodate four species: *B. eurasiaticogalactina* (Boidin & Lanq.) Leal-Dutra, Dentinger & G.W. Griff., *B. galactina* (Fr.) Leal-Dutra, Dentinger & G.W. Griff., *B. neogalactina* (Boidin & Lanq.) Leal-Dutra, Dentinger & G.W. Griff., and *B. octopodites* (Corner) Leal-Dutra, Dentinger & G.W. Griff. Stalpers et al. (2021) transferred two *Michenera* Berk. & M.A. species to *Scytinostroma* and treated the former genus as a synonym of the latter. Based on concatenated ITS1–5.8S–ITS2–nrLSU sequence data, Li et al. (2023) studied the phylogenetic analyses of *Peniophoraceae*, including samples of *Scytinostroma* s.s., and introduced four new species: *S. beijingensis*, *S. boidinii*, *S. subduriusculum*, and *S. subrenisporum*.

During investigations of wood-inhabiting fungi in Yunnan Province, China, many specimens were collected. To clarify the placement and relationships of these specimens, a phylogenetic and taxonomic study based on ITS and nrLSU sequences was conducted. These specimens were assigned to the genera *Gloeopeniophorella* and *Scytinostroma* within the order *Russulales*. Therefore, two new species, *G. luteola* and *S. tenuissimum*, are proposed with descriptions and illustrations, based on morphological characteristics and phylogenetic analyses. Additionally, the Pairwise Homoplasy Index (PHI) analyses were conducted among new species and similar taxa.

## Materials and methods

### Sample collection and herbarium specimen preparation

The fresh fruiting bodies were collected from dead bamboo in Yunnan Province, China. The samples were photographed in situ using a Nikon D7100 camera, and fresh macroscopic and collection details were recorded (Rathnayaka et al. 2025). All photos were focus-stacked using Helicon Focus software. Macroscopic details were recorded, and the samples were transported to a field station, where the fruiting bodies were dried using an electronic food dryer at 45 °C (Dong et al. 2024). Once dried, the specimens were sealed in envelopes and zip-lock plastic bags and labeled (Yang et al. 2025). The dried specimens were deposited in the Herbarium of the Southwest Forestry University (SWFC), Kunming, Yunnan Province, China.

### Morphology

The macro-morphological descriptions were based on field notes and photos collected in the field and in the laboratory. Petersen (1996) was followed for color terminology. The micromorphological data were obtained from dried specimens observed under a light microscope at 1000× oil immersion (Zhao et al. 2023; Dong et al. 2025). Sections were mounted in 5% KOH and 1% phloxine B (C_20_H_2_Br_4_Cl_4_Na_2_O_5_), and Cotton Blue and Melzer’s reagent were used where necessary to observe micromorphology, following the method of Deng et al. (2025). To present the variations of spore sizes, 5% of measurements were excluded from each end of the range and shown in parentheses. A minimum of 30 basidiospores from each specimen was measured. Stalks were excluded from basidia measurements, and the hilar appendage was excluded from basidiospore measurements. The MycoBank numbers were registered in the MycoBank database (http://www.mycobank.org).

The following abbreviations are used:

**CB** Cotton Blue

**CB+** cyanophilous

**CB–** acyanophilous

**IKI** Melzer’s reagent

**IKI–** both inamyloid and non-dextrinoid

**KOH** 5% potassium hydroxide water solution

***L*** mean spore length (arithmetic average for all spores)

***W*** mean spore width (arithmetic average for all spores)

***n*** a/b (number of spores (a) measured from the given number (b) of specimens)

***Q*** variation in the *L/W* ratios between the specimens studied

### Molecular phylogeny

The CTAB rapid fungi genome extraction kit-DN14 (Aidlab Biotechnologies Co., Ltd., Beijing) was used to obtain genomic DNA from dried specimens, according to the manufacturer’s instructions. The gene fragments employed in this study are detailed in Table 1.

**Table 1.** List of species, specimens, and GenBank accession numbers of sequences used in this study. Bold indicates new species. [* indicates type materials].

Species name	Sample no.	GenBank accession no.		Country	References
		ITS	nLSU		
*Acanthobasidium delicatum*	CBS 233.86	MH861948	MH873638	France	Vu et al. (2019)
*Acanthofungus rimosus*	Wu 9601-1*	MF043521	AY039333	China	Wu et al. (2019)
*Aleurobotrys botryosus*	He2712	KX306877	KY450788	China	Xu et al. (2025)
*Aleurocystidiellum subcruentatum*	HHB-17353-sp	KU559360	KU574818	USA	Xu et al. (2025)
*A. tsugae*	He 4024	KY706210	KY706222	China	Xu et al. (2025)
*Aleurodiscus gigasporus*	He 5605*	MW533084	MW528920	China	Xu et al. (2025)
*Amylostereum chailletii*	NH 8031	AF506406	AF506406	Sweden	Larsson and Larsson (2003)
*Asterostroma cervicolor*	He 4020	KY263860	KY263868	Japan	Suhara et al. (2010)
*A. laxum*	EL 33-99	AF506410	AF506410	Sweden	Larsson and Larsson (2003)
*Auriscalpium vulgare*	EL 33-95	AF506375	AF506375	Sweden	Larsson and Larsson (2003)
*Baltazaria galactina*	He 4999	MK625618	MK625547	France	Vu et al. (2019)
*B. neogalactina*	CBS 755.86	MH862037	MH873724	France	Vu et al. (2019)
*Bondarzewia montana*	CBS 372.59	MH857893	—	Germany	Vu et al. (2019)
*Conferticium subtropicum*	He 1827*	KY860405	KY860463	China	Xu et al. (2025)
*Confertobasidium olivaceoalbum*	FP 90196	AF511648	AF511648	—	Larsson and Larsson (2003)
*Confertotrama rugulosa*	He 3427	MW533086	MW528925	China	Xu et al. (2025)
*Dentipellis fragilis*	Dai 12550	JQ349110	JQ349096	China	Zhou and Dai (2013)
*Dentipratulum bialoviesense*	GG 1645	AF506389	AF506389	Sweden	Larsson and Larsson (2003)
*Dichostereum boidinii*	He 5026	MH538324	MH538330	China	Xu et al. (2025)
*Dichostereum pallescens*	CBS 719.81	MH861457	MH873199	USA	Vu et al. (2019)
*Echinodontium ryvardenii*	Ryvarden 43370	AF506431	AF506431	Italy	Chen et al. (2016)
*Gelatinostereum phlebioides*	He 6340	MW533095	MW528941	China	Xu et al. (2025)
*Gloeocystidiellum aspellum*	LIN 625	AF506432	AF506432	Sweden	Zhao and Zhao (2023)
*G. compactum*	Wu 880615-21	AF506434	AF506434	Sweden	Zhao and Zhao (2023)
*G. formosanum*	Wu 9404-19	AF506439	AF506439	Sweden	Zhao and Zhao (2023)
*G. porosum*	EB 990923	AY048881	AY048881	Sweden	Zhao and Zhao (2023)
*G. porosum*	FP 101749	AF310091	AF310091	Sweden	Zhao and Zhao (2023)
*Gloeocystidiopsis tenuissima*	He 3575*	KX306880	KY706214	China	Xu et al. (2025)
*Gloeomyces parvisporus*	Wu 1307–84	LC433897	LC433904	China	Xu et al. (2025)
*Gloeopeniophorella bambusicola*	CLZhao 35561	PP819702	—	China	Yang et al. (2025)
*G. convolvens*	KHL 10103	AF506435	AF506435	Sweden	Zhao and Zhao (2023)
*G. laxa*	He 4176	KY860413	KY860472	China	Zhao and Zhao (2023)
*G. laxa*	Wu 911010-8	AF506440	AF506440	Sweden	Zhao and Zhao (2023)
***G. luteola***	CLZhao 42933*	**PX712794**	**PX712799**	**China**	**Present study**
***G. luteola***	**CLZhao 48909**	**PX712795**	—	**China**	**Present study**
*Gloeosoma vitellinum*	646cc	MT831039	MT831019	Argentina	Rajchenberg et al. (2021)
*Gloiodon strigosus*	JS 26147	AF506449	AF506449	Norway	Chen et al. (2016)
*Gloiothele lamellosa*	CBS 404.83	MH861620	MH873337	Netherlands	Vu et al. (2019)
*G. torrendii*	JB 18615	AF506455	AF506455	Sweden	Larsson and Larsson (2003)
*Hericium americanum*	DAOM 21467	AF506458	AF506458	Sweden	Larsson and Larsson (2003)
*Heterobasidion annosum*	Dai 20962	ON417163	ON417213	China	Liu et al. (2019)
*Lachnocladium schweinfurthianum*	KM 49740	MH260033	MH260051	United Kingdom	Leal-Dutra et al. (2018)
*Lactarius leonis*	SJ 91016	AF506411	AF506411	Sweden	Zhao and Zhao (2023)
*L. torminosus*	CBS 197.72	MH860447	MH872175	Netherlands	Zhao and Zhao (2023)
*L. volemus*	LE 254509	JQ753937	JQ348388	Belgium	Zhao and Zhao (2023)
*L. taibaiensis*	HKAS 122860	OL423562	OL423575	China	Zhao and Zhao (2023)
*Lauriliella taiwanensis*	Wu 0808-116	KY172890	KY172905	China	Liu et al. (2017)
*Laxitextum bicolor*	Dai 14882	KY860394	KY860452	Sweden	Larsson and Larsson (2003)
*Lentinellus cochleatus*	KGN 960928	AF506417	AF506417	Sweden	Larsson and Larsson (2003)
*Megalocystidium leucoxanthum*	CBS 269.54	MH857325	MH868866	France	Vu et al. (2019)
*Metulodontia nivea*	NH 13108	AF506423	AF506423	Sweden	Larsson and Larsson (2003)
*Multifurca ochricompacta*	JJ 2010.08	MH063879	MH063844	USA	Zhao and Zhao (2023)
*M. orientalis*	XHW 3034	MH063856	MH063825	China	Zhao and Zhao (2023)
*M. pseudofurcata*	XHW 3205	MH063849	MH063819	China	Zhao and Zhao (2023)
*Peniophora incarnata*	NH 10271	AF506425	AF506425	Sweden	Larsson and Larsson (2003)
*P. quercina*	CBS 407.50	MH856687	MH868204	China	Vu et al. (2019)
*Russula aurantiaca*	SJ 93006	AF506427	AF506427	France	Zhao and Zhao (2023)
*R. emetica*	517IS76	AY061673	AY061673	USA	Zhao and Zhao (2023)
*R. nauseosa*	SJ97015	AF506462	AF506462	Sweden	Zhao and Zhao (2023)
*R. persicina*	SJ98044	AF506463	AF506463	Sweden	Zhao and Zhao (2023)
*Scytinostroma acystidiatum*	Dai 24608	OQ689127	OQ629351	China	Zhang et al. (2023)
*S. acystidiatum*	KUC 20121019-32	KJ668461	—	Korea	Jang et al. (2016)
*S. alutum*	CBS 762.81	MH861482	MH873221	France	Vu et al. (2019)
*S. alutum*	CBS 763.81	MH861483	MH873222	France	Vu et al. (2019)
*S. artocreas*	GHL-2016-Oct	MH142900	MH204691	USA	Liu et al. (2019)
*S. bambusinum*	JXH 596	OR510628	PP660873	China	Ji et al. (2024)
*S. bambusinum*	JXH 643	OR510627	PP660872	China	Ji et al. (2024)
*S. beijingensis*	He 7768	OQ731943	OQ729731	China	Li et al. (2023)
*S. boidinii*	He 5138	MK625572	MK625497	China	Li et al. (2023)
*S. boidinii*	He 6911	OQ731934	OQ729724	China	Li et al. (2023)
*S. caudisporum*	CBS 746.86	MH862030	NG073580	Gabon	Vu et al. (2019)
*S. crispulum*	CBS 716.86	MH862013	MH873703	Reunion	Vu et al. (2019)
*S. crispulum*	CBS 717.86	MH862014	MH873704	France	Vu et al. (2019)
*S. daweishanense*	CLZhao 17926 *	OR096194	OR461462	China	Dong et al. (2024)
*S. decidens*	CBS 714.86	MH862011	MH873701	France	Vu et al. (2019)
*S. decidens*	CBS 715.86	MH862012	MH873702	France	Vu et al. (2019)
*S. duriusculum*	CBS 757.81	MH861477	MH873216	France	Vu et al. (2019)
*S. duriusculum*	CBS 758.81	MH861478	MH873217	France	Vu et al. (2019)
*S. hemidichophyticum*	CBS 759.81	MH861479	MH873218	France	Vu et al. (2019)
*S. hemidichophyticum*	CBS 760.81	MH861480	MH873219	France	Vu et al. (2019)
*S. jacksonii*	CBS 239.87	MH862071	MH873759	Canada	Vu et al. (2019)
*S. macrospermum*	Dai 24606	OQ689126	OQ629350	China	Zhang et al. (2023)
*S. macrospermum*	M 2138	LC327052	—	Japan	Zhang et al. (2023)
*S. mediterraneense*	CBS 764.86	MH862045	MH873732	France	Vu et al. (2019)
*S. mediterraneense*	CBS 765.86	MH862046	MH873733	France	Vu et al. (2019)
*S. microspermum*	CBS 238.87	MH862070	—	Guadeloupe	Vu et al. (2019)
*S. ochroleucum*	CBS 767.86	MH862048	—	France	Vu et al. (2019)
*S. ochroleucum*	CBS 768.86	MH862049	MH873735	France	Vu et al. (2019)
*S. phaeosarcum*	CBS 761.81	MH861481	MH873220	Coted’Ivoire	Vu et al. (2019)
*S. portentosum*	CBS 503.48	MH856447	MH873220	Canada	Vu et al. (2019)
*S. pseudopraestans*	CBS 737.91	MH862322	MH873994	Netherlands	Vu et al. (2019)
*S. pseudopraestans*	CBS 738.91	MH862323	MH873995	Netherlands	Vu et al. (2019)
*S. quintasianum*	CBS 749.86	MH862031	MH873719	Netherlands	Vu et al. (2019)
*S. quintasianum*	CBS 750.86	MH862032	MH873720	Netherlands	Vu et al. (2019)
*S. renisporum*	CBS 771.86	MH862051	MH873738	Bali	Vu et al. (2019)
*S. renisporum*	CBS 772.86	MH862052	MH873739	Bali	Vu et al. (2019)
*S. subduriusculum*	He 3590	MK625571	MK625499	China	Li et al. (2023)
*S. subduriusculum*	He 4146	MK625570	MK625498	China	Li et al. (2023)
***S. tenuissimum***	CLZhao 37325*	**PX712796**	**PX712798**	**China**	**Present study**
***S. tenuissimum***	**CLZhao 48871**	**PX712797**	—	**China**	**Present study**
*S. yunnanense*	CLZhao 10758	MT611445	—	China	Wang et al. (2020)
*S. yunnanense*	CLZhao 10802	MT611446	—	China	Wang et al. (2020)
*Sistotrema brinkmannii*	NH 11412	AF506473	AF506473	Sweden	Larsson and Larsson (2003)
*S. coronilla*	RAS538 SV2	OR471183	OR470996	USA	Swenie et al. (2023)
*S. muscicola*	KHL 8791	AF506474	AF506474	Sweden	Zhao and Zhao (2023)
*Stereodiscus antarcticus*	MR 11265	MT831048	MT831028	Argentina	Rajchenberg et al. (2021)
*Stereum hirsutum*	He 3504	MK625629	MK625557	China	Unpublished
*Terrestriporia alba*	Dai 18548	MT068564	MT068560	Malaysia	Wu et al. (2020)
*T. alba*	Dai 18556	MT068565	MT068561	Malaysia	Wu et al. (2020)
*Vararia breviphysa*	CBS 643.81	MH873144	MH873144	Gabon	Vu et al. (2019)
*V. gomezii*	CBS 661.81	MH873154	MH873154	France	Vu et al. (2019)
*V. sigmatospora*	CBS 748.91	MH874001	MH874001	Netherlands	Vu et al. (2019)
*V. sinensis*	CLZhao 25160*	OR102494	OR510678	China	Deng et al. (2024)
*V. trinidadensis*	CBS 650.84	MH873495	MH873495	Madagascar	Vu et al. (2019)
*Xylobolus frustulatus*	He 2231	MW263995	MW263979	China	Xu et al. (2025)

The PCR protocol for ITS was as follows: initial denaturation at 95 °C for 3 min, followed by 35 cycles at 94 °C for 40 s and 58 °C for 40 s. The PCR protocol for nrLSU was as follows: initial denaturation at 94 °C for 1 min, followed by 35 cycles at 94 °C for 30 s, 48 °C for 1 min, and 72 °C for 1.5 min, and a final extension at 72 °C for 10 min (Dong et al. 2025). The PCR products were purified and sequenced at Kunming Tsingke Biological Technology Limited Company, Kunming, Yunnan Province, P.R. China. All newly generated sequences were deposited in GenBank (Table 1).

Maximum likelihood analyses were performed using the CIPRES Science Gateway (https://www.phylo.org/portal2/login!input.action; Miller et al. 2012) based on the dataset using the RAxML-HPC BlackBox tool, with RAxML halt bootstrapping automatically, 0.25 for maximum hours, and obtaining the best tree using ML search. Other parameters in ML analysis used default settings, and statistical support values were obtained using nonparametric bootstrapping with 1000 replicates. Bayesian inference (BI) analysis was performed on the same dataset using MrBayes v3.2.7a (Ronquist et al. 2012). The best substitution model for the dataset was selected using ModelFinder v2.2.0 (Kalyaanamoorthy et al. 2017) with the Bayesian Information Criterion, and the model was used for Bayesian analysis. Four Markov chains were run from random starting trees. Trees were sampled every 1000^th^ generation. The first 25% of sampled trees were discarded as burn-in, while the remaining trees were used to construct a 50% majority consensus tree and to calculate Bayesian posterior probabilities (BPPs).

Phylogenetic trees were visualized and adjusted using FigTree v1.4.4 (http://tree.bio.ed.ac.uk/software/figtree), and the exports were edited using Adobe Illustrator CS6 software (Adobe Systems, USA). Branches of the consensus tree that received bootstrap support for maximum likelihood (ML) equal to or above 70%, and Bayesian inference (BI) equal to or above 0.95, respectively, were considered significant.

## Results

### Molecular phylogeny

**Figure 1.**
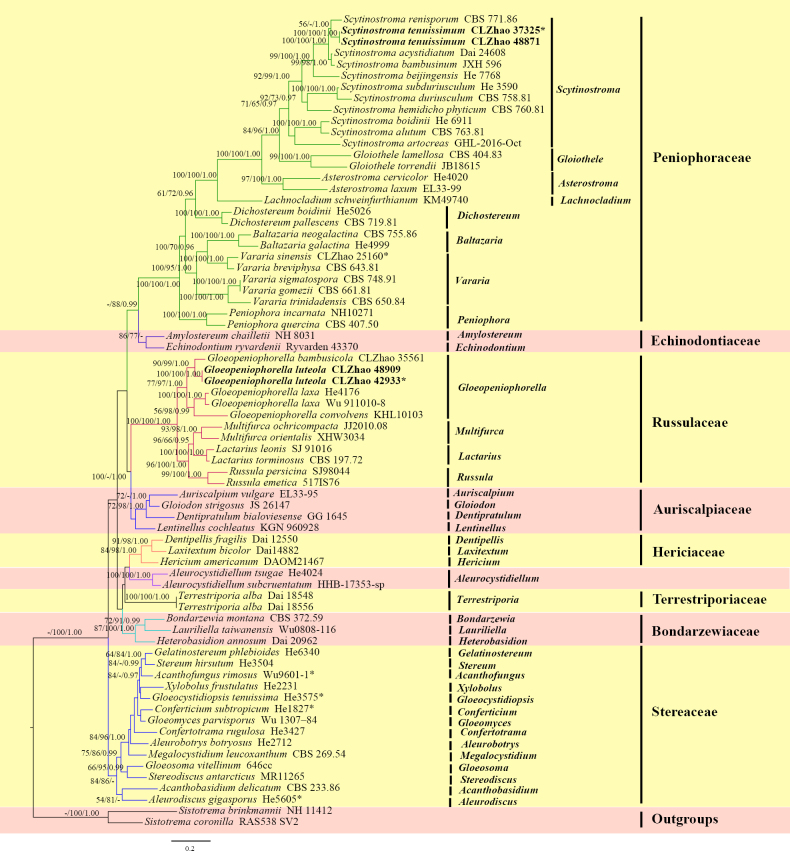
Maximum likelihood strict consensus tree illustrating the phylogeny of two new species within the order *Russulales*, based on the ITS + nrLSU sequence dataset. Branches are labeled with maximum parsimony bootstrap values equal to or higher than 50%, maximum likelihood bootstrap values equal to or above 70% and Bayesian posterior probabilities equal to or above 0.9. The new species are in bold. * refers to type material and holotype.

The ITS + nLSU dataset (Fig. 1) comprised sequences from 72 fungal specimens representing 68 taxa. The dataset had an aligned length of 2380 characters, of which 1,194 were constant, 230 were variable and parsimony-uninformative, and 956 (50%) were parsimony-informative. Maximum parsimony analysis yielded three equally parsimonious trees (TL = 7132, CI = 0.3100, HI = 0.6900, RI = 0.5889, and RC = 0.1826). The best nucleotide model for the ITS + nLSU dataset, estimated and applied in the Bayesian analysis, was TIM2+I+G. Bayesian and ML analyses yielded a topology similar to that of the MP analyses. The Bayesian analysis had an average standard deviation of split frequencies = 0.009571 (BI), and the effective sample size (ESS) across the two runs is double the average ESS (avg. ESS) = 354. The phylogram based on the ITS + nLSU rDNA gene regions (Fig. 1) included eight families within the order *Russulales*: *Auriscalpiaceae*, *Bondarzewiaceae*, *Echinodontiaceae*, *Hericiaceae*, *Peniophoraceae*, *Russulaceae*, *Stereaceae*, and *Terrestriporiaceae*. Phylogenetic analysis based on the concatenated ITS + nLSU dataset demonstrated that the two new species grouped within the order *Russulales*, in which *G. luteola* grouped into the family *Russulaceae*, while *Scytinostroma tenuissimum* grouped into the family *Peniophoraceae*.

**Figure 2.**
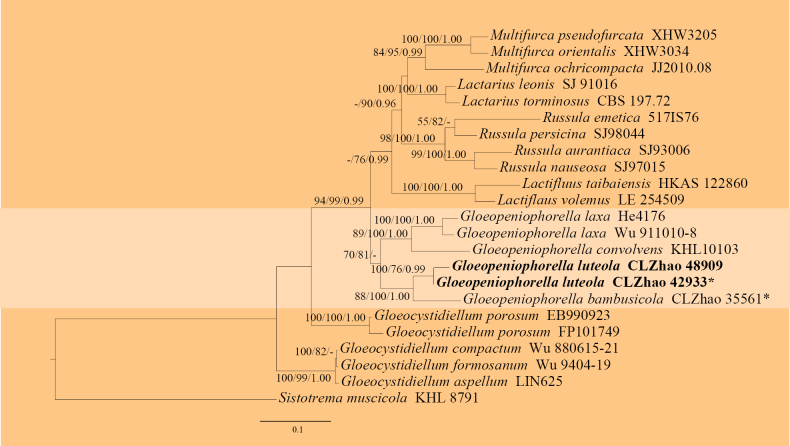
Maximum likelihood strict consensus tree illustrating the phylogeny of *Gloeopeniophorella luteola* and related species in the genus *Gloeopeniophorella*, based on the ITS + nrLSU dataset. Branches are labeled with maximum parsimony bootstrap values equal to or higher than 50%, maximum likelihood bootstrap values equal to or above 70% and Bayesian posterior probabilities equal to or above 0.9. The new species are in bold. * refers to type material and holotype.

The ITS + nLSU dataset (Fig. 2) comprised sequences from 26 fungal specimens representing 20 taxa. The dataset had an aligned length of 2,129 characters, of which 1,399 characters were constant, 267 were variable and parsimony-uninformative, and 463 were parsimony-informative. Maximum parsimony analysis yielded three equally parsimonious trees (TL = 1685, CI = 0.6421, HI = 0.3579, RI = 0.6350, and RC = 0.4077). The best nucleotide model for the ITS + nLSU dataset, estimated and applied in the Bayesian analysis, was TIM1ef+I+G. Bayesian and ML analyses resulted in a topology similar to that of the MP analysis. The Bayesian analysis had an average standard deviation of split frequencies = 0.009088 (BI), and the effective sample size (ESS) across the two runs is double the average ESS (avg. ESS) = 302. The phylogram based on the ITS + nLSU rDNA gene regions (Fig. 2) highlighted that the new species *Gloeopeniophorella luteola* was sister to *G. bambusicola* Yang, Yang & C.L. Zhao.

**Figure 3.**
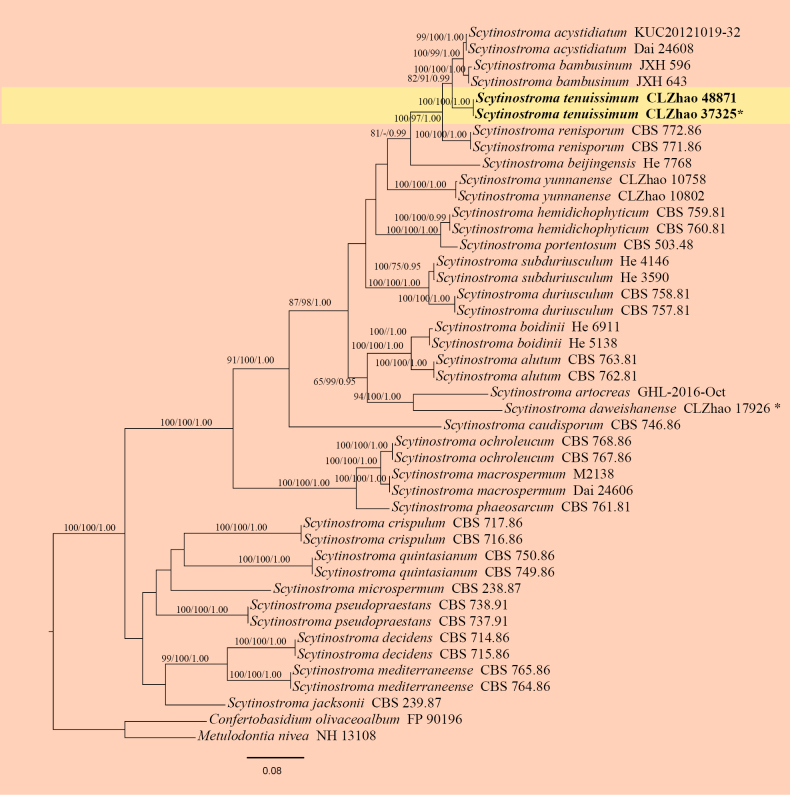
Maximum likelihood strict consensus tree illustrating the phylogeny of *Scytinostroma tenuissimum* and related species in the genus *Scytinostroma*, based on the ITS + nrLSU dataset. Branches are labeled with maximum parsimony bootstrap values equal to or higher than 50%, maximum likelihood bootstrap values equal to or above 70% and Bayesian posterior probabilities equal to or above 0.9. The new species are in bold. * refers to type material and holotype.

The ITS + nLSU dataset (Fig. 3) comprised sequences from 44 fungal specimens representing 27 taxa. The dataset had an aligned length of 2,215 characters, of which 1,366 characters were constant, 126 were variable and parsimony-uninformative, and 723 were parsimony-informative. Maximum parsimony analysis yielded three equally parsimonious trees (TL = 3222, CI = 0.4699, HI = 0.5301, RI = 0.7493, and RC = 0.3521). The best nucleotide model for the ITS + nLSU dataset, estimated and applied in the Bayesian analysis, was TIM2+I+G. Bayesian and ML analyses resulted in a topology similar to that of the MP analysis. The Bayesian analysis had an average standard deviation of split frequencies = 0.008826 (BI), and the effective sample size (ESS) across the two runs is double the average ESS (avg. ESS) = 160. The phylogram based on the ITS + nLSU rDNA gene regions (Fig. 3) indicated that the new species *Scytinostroma tenuissimum* grouped with *S. acystidiatum* Q.Y. Zhang, L.S. Bian & Qian Chen and *S. bambusinum* X.H. Ji.

### Taxonomy

#### Gloeopeniophorella luteola

Taxon classificationFungiRussulalesRussulaceae

C.L. Zhao sp. nov.

4DBB605C-82BF-5A3A-B293-6B14107C069E

862909

Figs 4, 5, 6, 7

##### Diagnosis.

It is characterized by its membranaceous, thin basidiomata with smooth hymenial surface, a monomitic hyphal system with simple septa, generative hyphae and gloeocystidia, thick-walled basidiospores measuring 4–4.9 × 3.8–4.4 µm.

**Figure 4.**
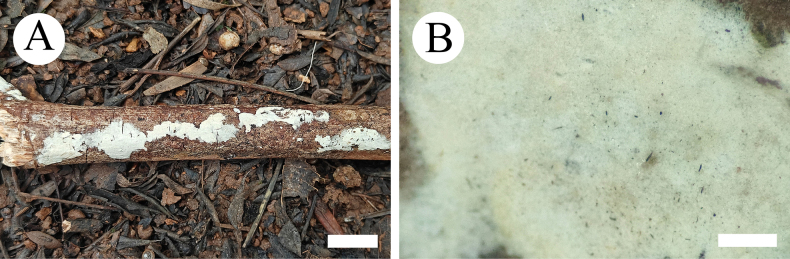
Basidiomata of *Gloeopeniophorella luteola* (CLZhao 42933, holotype). **A**. Basidiomata on the substrate; **B**. Hymenophore. Scale bars: 1 cm (**A**); 1 mm (**B**).

**Figure 5.**
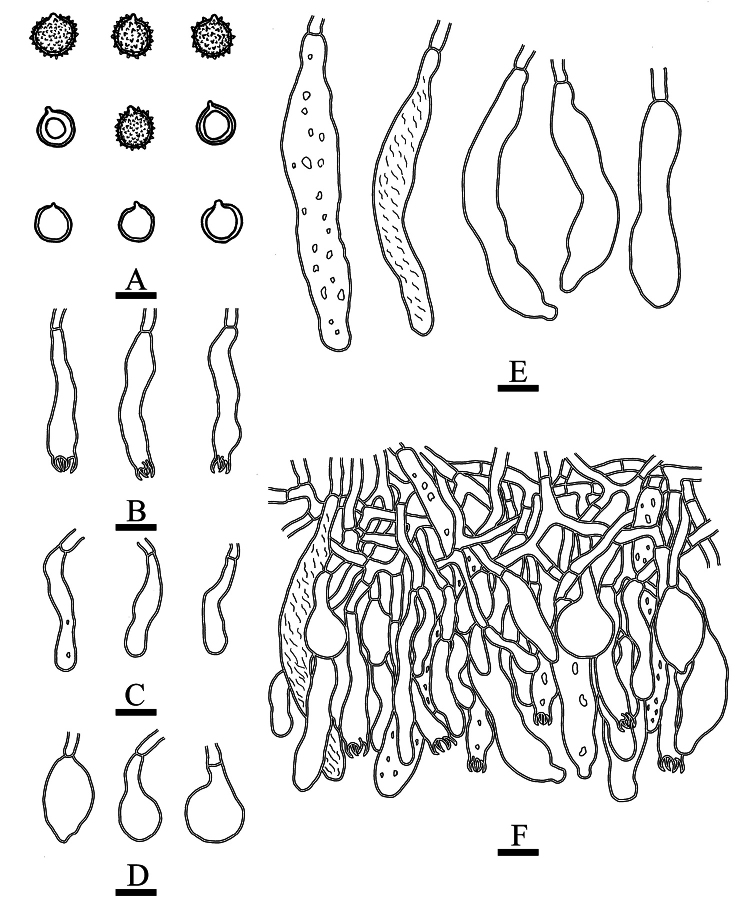
Microscopic structures of *Gloeopeniophorella luteola* (CLZhao 42933, holotype). **A**. Basidiospores; **B**. Basidia; **C**. Basidioles; **D**. Clavate gloeocystidia; **E**. Cylindrical gloeocystidia; **F**. Part of the vertical section of the hymenium. Scale bars: 5 µm (**A**); 10 µm (**B–F**).

**Figure 6.**
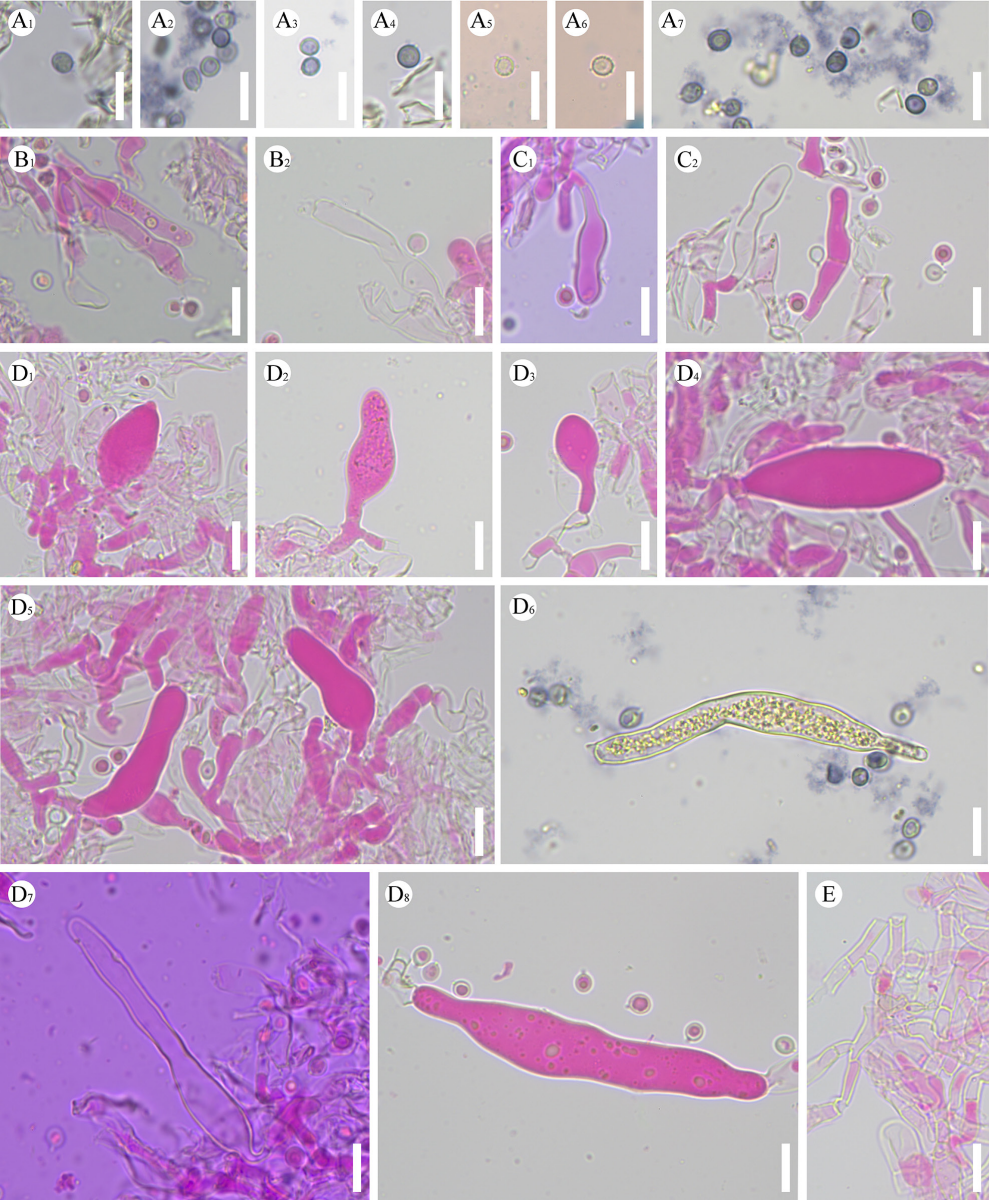
Sections of hymenium of *Gloeopeniophorella luteola* (CLZhao 42933, holotype). **A1–A7**. Basidiospores; **B1, B2**. Basidia; **C1, C2**. Basidioles; **D1–D8**. Gloeocystidia; **E**. Generative hyphae. Scale bars: 10 µm (**A–E**); 10 × 100 oil.

**Figure 7.**
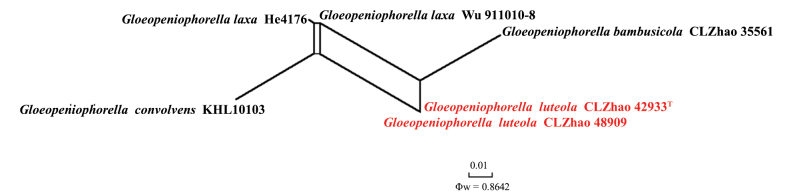
Split graphs showing the results of the PHI test for the ITS data of *Gloeopeniophorella luteola* and closely related taxa using LogDet transformation and splits decomposition. PHI test results Φw ≤ 0.05 indicate significant recombination within the dataset. New taxa are in red.

##### Holotype.

China • Yunnan Province, Dehong, Ruili City, Tongbiguan Provincial Nature Reserve, GPS coordinates 23°38'N, 97°51'E, altitude 1500 m asl., on the fallen angiosperm branches, leg. C.L. Zhao, 24 November 2024, CLZhao 42933 (SWFC 00042933).

##### Etymology.

luteola (Lat.) refers to the buff hymenial surface of the type specimens.

##### Basidiomata.

Basidiomata annual, resupinate, membranaceous, without odor or taste when fresh, up to 7.5 cm long, 1 cm wide, and up to 150 µm thick. Hymenial surface smooth, white to cream when fresh, cream to buff upon drying. Sterile margin white, thinning out, up to 1 mm wide.

##### Hyphal system.

Hyphal system monomitic; generative hyphae bearing simple septa, slightly thick-walled, colorless, all hyphae occasionally branched, flexuous, 2.5–3.5 µm in diameter, IKI–, CB–; tissues unchanged in KOH.

***Hymenium***. Gloeocystidia two types: 1) clavate to subglobose, colorless, smooth, thick-walled, 11–28.5 × 6–13 µm; 2) fusiform to cylindrical, abundant, smooth, colorless, thick-walled, basally inflated and slightly tapering towards the apices, filled with some refractive matter, 28–90.5 × 7.5–13.5 µm. Basidia cylindrical, colorless, thick-walled, with four sterigmata and a basal simple septum, 24–36 × 5–7 µm, basidioles in shape similar to basidia, but slightly smaller.

***Basidiospores***. Basidiospores globose, colorless, thick-walled, smooth to verrucose, IKI+, CB–, (3.8–)4–4.9(–5) × (3.4–)3.8–4.4(–4.8) µm, *L* = 4.44 µm, *W* = 4.09 µm, *Q* = 1.08–1.09 (*n* = 60/2).

##### Additional specimen examined (paratype).

China • Yunnan Province, Dehong, Ruili City, Tongbiguan Provincial Nature Reserve, GPS coordinates 23°38'N, 97°51'E, altitude 1500 m asl., on the fallen angiosperm branches, leg. C.L. Zhao, 5 November 2025, CLZhao 48909 (SWFC 00048909).

The application of the PHI test to the ITS tree-locus sequences revealed no evidence of recombination among phylogenetically related species. No significant recombination events were observed among *Gloeopeniophorella luteola* and phylogenetically closely related species (Fig. 7). The test results of the ITS sequence dataset show Φw = 0.8642 (Φw > 0.05) and that no recombination is present in the three new species with *G. luteola*.

#### Scytinostroma tenuissimum

Taxon classificationFungiRussulalesPeniophoraceae

C.L. Zhao sp. nov.

431A3D2E-A2A0-5F73-AEA8-633108442FDD

862911

Figs 8, 9, 10, 11

##### Diagnosis.

It is characterized by its soft membranaceous basidiomata with cream to pale pink hymenial surface, a dimitic hyphal system with clamped generative hyphae and ellipsoid basidiospores measuring 10.1–13.2 × 7.1–9.3 µm.

**Figure 8.**
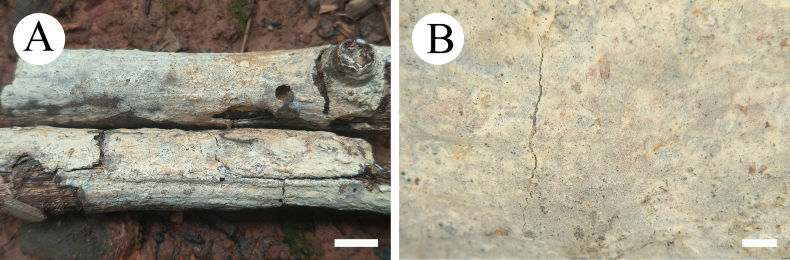
Basidiomata of *Scytinostroma tenuissimum* (CLZhao 37325, holotype). **A**. Basidiomata on the substrate; **B**. Hymenophore. Scale bars: 1 cm (**A**), 1 mm (**B**).

**Figure 9.**
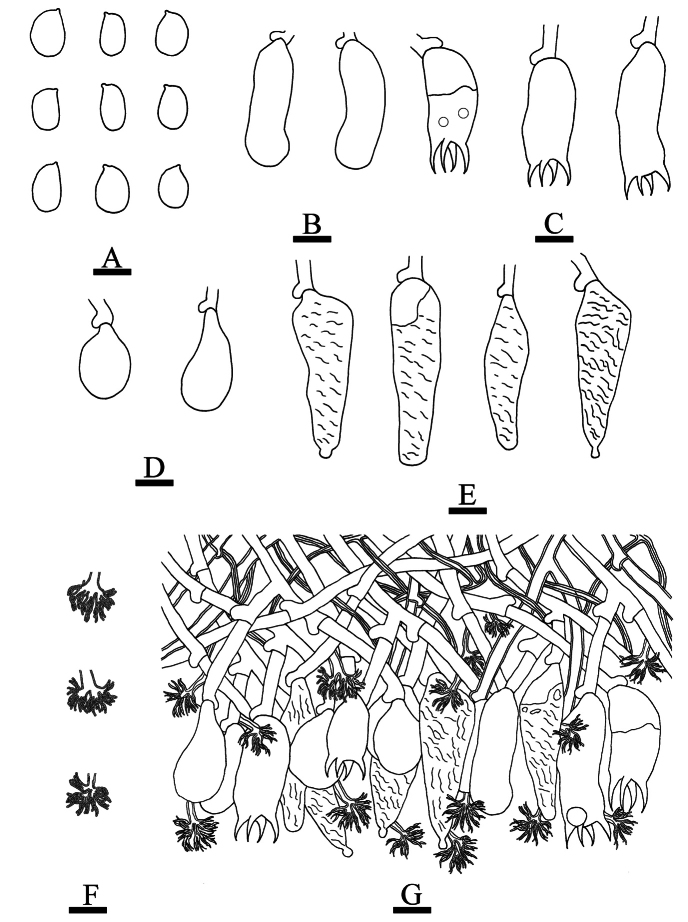
Microscopic structures of *Scytinostroma tenuissimum* (CLZhao 37325, holotype). **A**. Basidiospores; **B**. Basidia; **C**. Basidioles; **D**. Subglobose gloeocystidia; **E**. Fusiform gloeocystidia; **F**. Dendrohyphae; **G**. Part of the vertical section of the hymenium. Scale bars: 10 µm (**A–G**).

**Figure 10.**
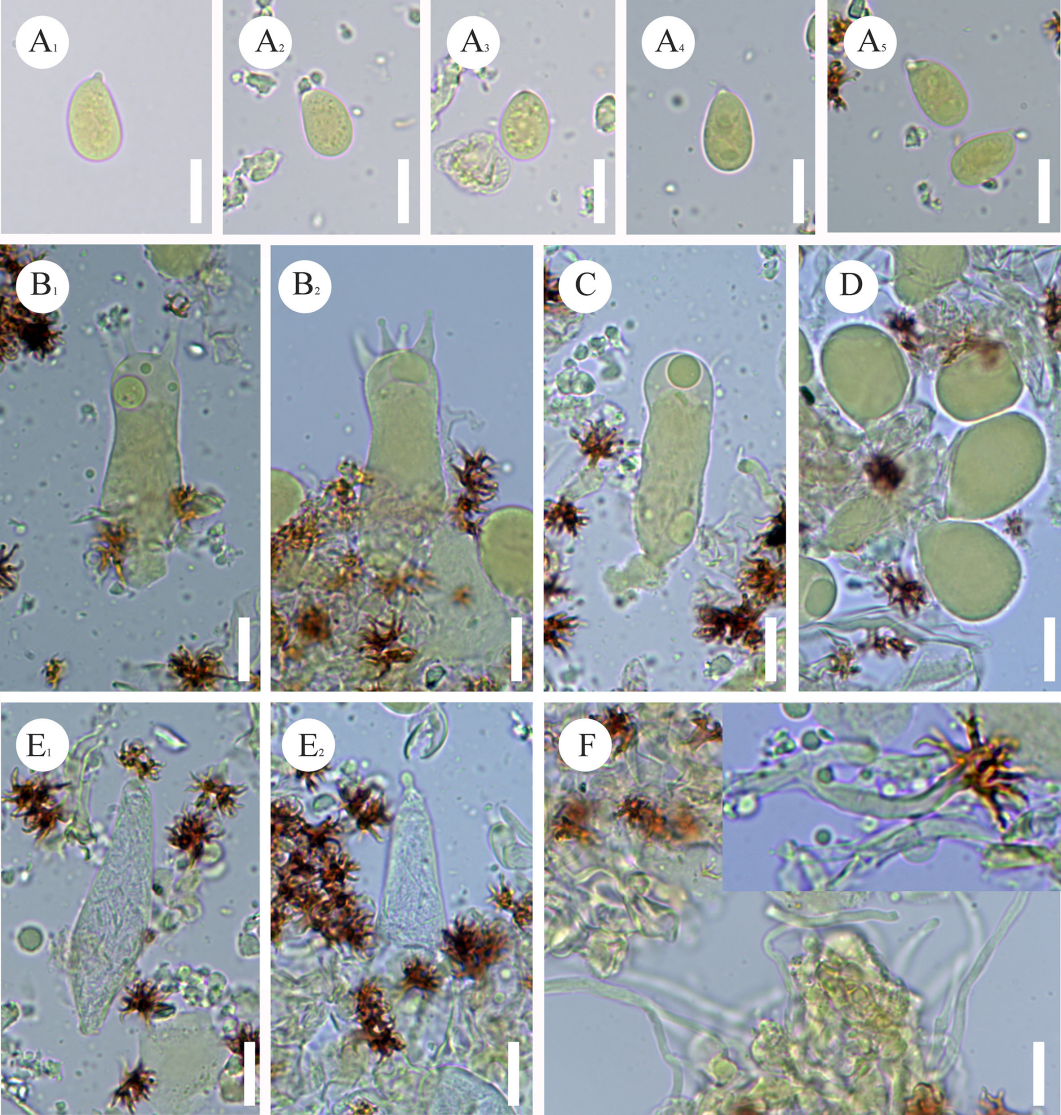
Sections of hymenium of *Scytinostroma tenuissimum* (CLZhao 37325, holotype). **A1–A5**. Basidiospores; **B1, B2**. Basidia; **C**. Basidioles; **D**. Globose cystidia; **E1, E2**. Gloeocystidia; **F**. A section of hyphae. Scale bars: 10 µm (**A–F**); 10 × 100 oil.

**Figure 11.**
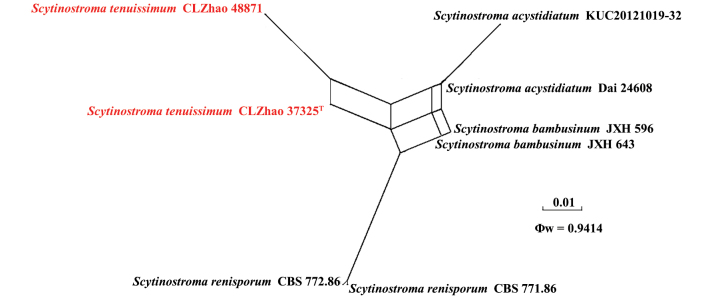
Split graphs showing the results of the PHI test for the ITS data of *Scytinostroma tenuissimum* and closely related taxa using LogDet transformation and splits decomposition. PHI test results Φw ≤ 0.05 indicate that there is significant recombination within the dataset. New taxa are in red.

##### Holotype.

China • Yunnan Province, Dehong, Yingjiang County, Tongbiguan Provincial Nature Reserve, GPS coordinates 24°71'N, 94°52'E, altitude 1500 m asl., on the fallen angiosperm branch, leg. C.L. Zhao, 2 July 2024, CLZhao 37325 (SWFC 00037325).

##### Etymology.

tenuissimum (Lat.) refers to the thin basidiomata of the type specimens.

##### Basidiomata.

Basidiomata annual, resupinate, membranaceous, thin, without odor or taste when fresh, fragile upon drying, up to 12 cm long, 1.5 cm wide, and up to 150 µm thick. Hymenial surface smooth, slightly creamy when fresh, cream to pale pink upon drying. Sterile margin slightly cream, thinning out, up to 1 mm wide.

##### Hyphal system.

Hyphal system dimitic; generative hyphae with clamp connections, thin- to slightly thick-walled, colorless, all hyphae occasionally branched, flexuous, 2–4.5 µm in diameter, IKI–, CB–; tissues unchanged in KOH. Skeletal hyphae densely branched, smooth, thick-walled, 2–4 µm in diameter, dendrophiles, strongly dextrinoid, capillary, irregularly branched with main branches and acute tips, smooth, thick-walled.

***Hymenium***. Gloeocystidia two types: 1) subglobose, colorless, smooth, thin-walled, 12–23 × 10–15 µm; 2) fusiform, abundant, smooth, colorless, thin-walled, basally inflated and slightly tapering towards the apices, filled with some refractive matter, 25.5–56 × 10.5–16 µm. Basidia barreled, colorless, thin-walled, with four sterigmata and a basal clamp connection, 25–36 × 9.8–14.3 µm, basidioles in shape similar to basidia, but slightly smaller.

***Basidiospores***. Basidiospores ellipsoid, colorless, thin-walled, smooth, IKI–, CB–, (9.4–)10.1–13.2(–13.7) × (7.0–)7.1–9.3(–9.7) µm, *L* = 11.51 µm, *W* = 7.95 µm, *Q* = 1.39–1.49 (*n* = 60/2).

##### Additional specimen examined (paratype).

China • Yunnan Province, Dehong, Yingjiang County, Tongbiguan Provincial Nature Reserve, GPS coordinates 24°71'N, 94°52'E, altitude 1500 m asl., on the fallen angiosperm branch, leg. C.L. Zhao, 2 July 2024, CLZhao 48871 (SWFC 00048871)

The application of the PHI test to the ITS tree-locus sequences revealed no evidence of recombination among phylogenetically related species. No significant recombination events were observed among *Scytinostroma tenuissimum* and phylogenetically closely related species (Fig. 11). The test results for the ITS sequence dataset show that Φw = 0.9414 (Φw > 0.05), indicating no recombination among the three new species and *S. tenuissimum*.

## Discussion

The family *Russulaceae* is considered an iconic lineage of mostly mushroom-forming basidiomycetes due to its importance as edible mushrooms in many parts of the world and its ubiquity as ectomycorrhizal symbionts in both temperate and tropical forested biomes (Looney et al. 2018). They display great variation in basidioma morphology, including erect and effused forms and gilled and nongilled forms. Earlier studies have shown these taxa to be related, and the group has been named the russuloid clade. In the present study, two new wood-inhabiting fungal species, *Gloeopeniophorella luteola* and *Scytinostroma tenuissimum*, are described based on phylogenetic analyses and morphological characteristics.

Phylogenetic relationships among russuloid basidiomycetes were investigated using sequence data from the nuclear 5.8S, ITS2, and large-subunit rDNA genes, yielding 13 major, well-supported clades within the russuloid clade (Larsson and Larsson 2003). In the present study, the phylogram based on the ITS + nLSU rDNA gene regions highlighted that the new species *Gloeopeniophorella luteola* was sister to *G. bambusicola*. However, morphologically, *G. bambusicola* differs from *G. luteola* by farinaceous basidiomata and clavate and thin-walled basidia; in addition, *G. bambusicola* grows on dead bamboo (Yang et al. 2025). The phylogenetic tree (ITS + nLSU rDNA gene) indicated that the new species *Scytinostroma tenuissimum* grouped with *S. acystidiatum* and *S. bambusinum*. However, morphologically, *S. acystidiatum* can be distinguished from *S. tenuissimum* by its smooth to locally tuberculate hymenial surface, simple-septate generative hyphae, distinctly smaller basidiospores (4.7–7 × 3.5–7 µm vs. 10.1–13.2 × 7.1–9.3 µm), and lack of cystidia (Zhang et al. 2023). The species *S. bambusinum* differs from *S. tenuissimum* by simple-septate generative hyphae, distinctly smaller basidiospores (5.5–7 × 4–5.3 µm vs. 10.1–13.2 × 7.1–9.3 µm), and growth on dead bamboo (Ji et al. 2024).

Fungi constitute one of the most diverse groups of organisms on Earth and play a critical role in ecosystem processes and functions (Hibbett et al. 2025). Recent advances in DNA sequencing techniques have transformed the study of fungal taxonomy and diversity, resulting in the formal description of approximately 160,000 species to date (Hibbett et al. 2025). Wood-inhabiting fungi represent a well-studied group within Basidiomycota, encompassing various poroid, smooth, grandinoid, odontioid, and hydnoid basidiomata in China (Wu et al. 2022; Dong et al. 2024; Ghobad-Nejhad et al. 2024; Liu et al. 2025; Jerusalem et al. 2025; Koga et al. 2025; Song et al. 2025; Wijesinghe et al. 2025; Zhao et al. 2025). In recent years, numerous corticioid species have been reported and described within the order *Russulales* (Deng et al. 2024; Wang et al. 2025a, b; Zhu et al. 2025). However, the corticioid diversity of *Russulales* remains insufficiently documented, particularly in subtropical and tropical regions of China. Continued field surveys, combined with integrative morphological and molecular approaches, are expected to reveal additional undescribed corticioid taxa, and future collections may further uncover species of *Russulales*.

## Supplementary Material

XML Treatment for Gloeopeniophorella luteola

XML Treatment for Scytinostroma tenuissimum

## Additional information

### Artificial Intelligence (AI) use

The authors accept full responsibility for the content of the manuscript, including the disclosure of any use of AI.

No AI tools were used in the preparation of this manuscript.

### Author ORCIDs

C. Zhao https://orcid.org/0000-0002-8668-1075

Q. Li https://orcid.org/0009-0007-4990-9273

X. Liu https://orcid.org/0000-0003-0100-2094

### Data availability

All of the data that support the findings of this study are available in the main text.
